# Suggestion for new 4.4 mm pubo-femoral distance cut-off value for hip instability in lateral position during DDH screening

**DOI:** 10.1080/17453674.2018.1554404

**Published:** 2018-12-10

**Authors:** Hans-Christen Husum, Michel B Hellfritzsch, Nina Hardgrib, Bjarne Møller-Madsen, Ole Rahbek

**Affiliations:** a Department of Children’s Orthopaedics, Aarhus University Hospital, Aarhus; b Department of Radiology, Aarhus University Hospital, Aarhus;; c Department of Paediatrics, Aarhus University Hospital, Aarhus, Denmark

## Abstract

Background and purpose — Current selective screening algorithms for developmental dysplasia of the hip (DDH) are insufficient. Universal screening programs have been proposed but so far have been deemed too expensive and time consuming. The pubo-femoral distance may solve this problem as a quick, low-cost, highly sensitive, and specific sonographic measurement for DDH, but this has only been validated in the supine position. Therefore we validated pubo-femoral distance (PFD) in the lateral position as an indicator for instability of the hip.

Methods — All participants had undergone ultrasonographic diagnostics using the modified Graf technique. In addition, PFD measurements in lateral position were performed. Results were compared between 25 infants who had been treated for DDH because of dysplastic appearance on ultrasound combined with clinical instability and a control group consisting of 100 untreated infants screened for DDH. Sensitivity, specificity, and cut-off points were determined using Receiver operating characteristics (ROC) analysis.

Results — We found a mean PFD of 6.8 mm (6.2–7.4) in the treated group with a control group PFD of 3.4 mm (3.3–3.6) (p < 0.005). A PFD value above a threshold of 4.4 mm yielded a sensitivity of 100% and a specificity of 93% for detecting unstable DDH.

Interpretation — PFD measured in lateral position was statistically significantly increased in hips of children treated for DDH with Denis Browne hip brace compared with healthy children with unaffected stable hips. Furthermore, the PFD measurement had a high level of sensitivity and specificity at a cut-off value of 4.4 mm. A cut-off value of 6.00 mm has previously been reported as the gold standard in supine position. We suggest that 4.4 mm is used in lateral position.

Universal ultrasound screening decreases the rate of late diagnosis and surgical interventions, and is cost-effective (Thaler et al. 2011) as well as useful in detecting developmental dysplasia of the hip (DDH) in children with no apparent clinical symptoms or risk factors (Marks et al. 1994).

In Denmark universal ultrasound screening is not implemented. Infants are routinely screened for hip instability by a midwife after birth, and at a 5-week routine follow-up at their general practitioner. The screening is clinical and is based primarily on the Barlow and Ortolani maneuvers. If there are positive clinical findings, the child is referred to the next level of the screening program, which is an ultrasound by an experienced radiologist for further diagnostics as clinical examination alone is not considered sufficient (Rosenberg et al. 1998, Roposch et al. 2006).

Children with established risk factors for the development of DDH (breech presentation, 1 degree relative to DDH, oligohydramnios, congenital deformities), bypass the general screening program, and are referred to ultrasound examination directly by the midwife. These infants are screened for DDH with a combination of clinical examination of the hip instability and ultrasound using a modified Graf technique (Graf 1983). However, these gold standard ultrasound examinations require a skilled radiologist in order to obtain a correct diagnosis of DDH (Hell 2008, Omeroğlu et al. 2001).

Treguier et al. (2013) have implemented the pubo-femoral distance (PFD) measured in supine position, as developed by Couture et al. (2011), in a screening program in France. This is a simple measurement to detect hip instability and identify at-risk hips where early intervention is warranted.

The PFD method does not require an experienced radiologist; it can be used by radiologists or technicians with limited experience in ultrasound. This could potentially reduce the need for skilled radiologists to be involved in the early stage of screening and facilitate implementation of a universal ultrasound screening program. The PFD method has been demonstrated by Teixeira et al. (2015) to be highly sensitive and specific in the diagnosis of DDH at a cut-off point of 4.6 mm with the infant in lateral position. In contrast Treguier et al. (2013) use a cut-off point of 6.0 mm when measuring the infant in supine position and the hip flexed in adduction. The cut-off point for PFD may therefore be dependent on position of the patient and the ultrasonic plane as the reported thresholds are conflicting. Furthermore, the method has not to our knowledge been validated in a Danish population.

We validated PFD as a tool for early diagnosis of DDH in Danish children by evaluating the PFD measurements of children diagnosed with, and treated for, unstable DDH on the basis of Graf’s method combined with clinical stability testing. The sensitivity and specificity of the method is examined, and an optimal cut-off point for the PFD measurement in lateral position in the diagnosis of instable DDH is determined.

## Methods

### Cases

We identified 159 patients examined sonographically for DDH and subsequently treated with a Denis Browne (DB) hip brace for unilateral or bilateral unstable DDH at Aarhus University Hospital from February 2013 to September 2016. All treatments with the DB hip brace are coded and registered systematically with the treatment code BLPD10-13 in the hospital system. The identified patients have been validated against the radiological PACS reports to ensure the correct diagnosis. The total number of patients treated was 41.

Patients were excluded if PFD measurements were missing (n =17). This was due to dislocation of the hip making the PFD immeasurably high (n = 1) or patients who had been referred from hospitals with different ultrasound examination algorithms and therefore no recorded PFD values (n = 3).

**Figure 1.**
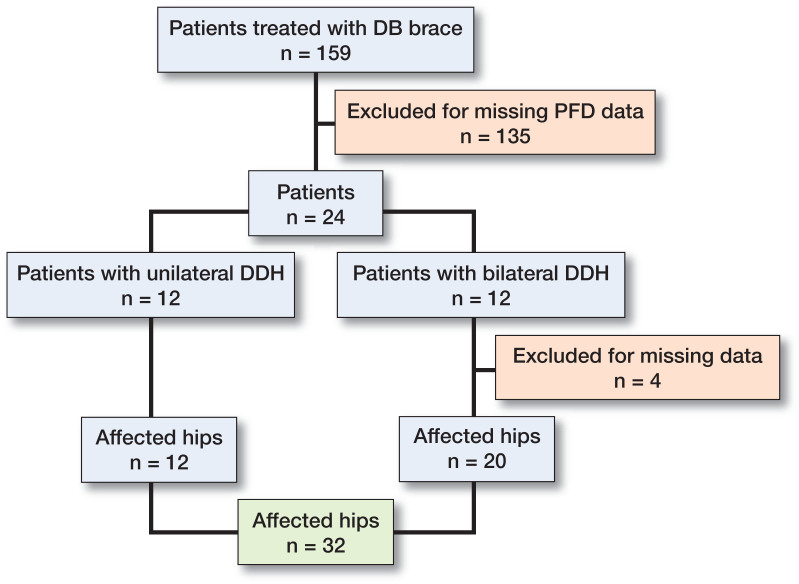
Recruitment process: flowchart of cases with hip instability.

A total of 24 patients (23 girls) were included, 12 with unilateral DDH and 12 with bilateral DDH, in all 32 affected hips (Figure 1) (4 hips in the bilateral group were missing PFD values). Mean age at the time of referral to initial ultrasound screening was 25 days.

Radiological charts were reviewed for assessing the Graf measurements, bone rim percentage (BRP), and PFD values.

### Controls

The healthy control group consists of all patients referred for an ultrasound examination at the same department in the period January 2017 to March 2017, whose ultrasound examination was deemed normal using the modified Graf technique. No patients were excluded from the control group. 100 patients had both hips examined sonographically with a normal result, in the period January 2017 to March 2017 in our department, totaling 200 hips examined, of which 95 were female and 103 were male, and mean age at referral was 39 days. PFD values were available on 198 hips, making up our control group.

### Ultrasound examination

**Figure 2.**
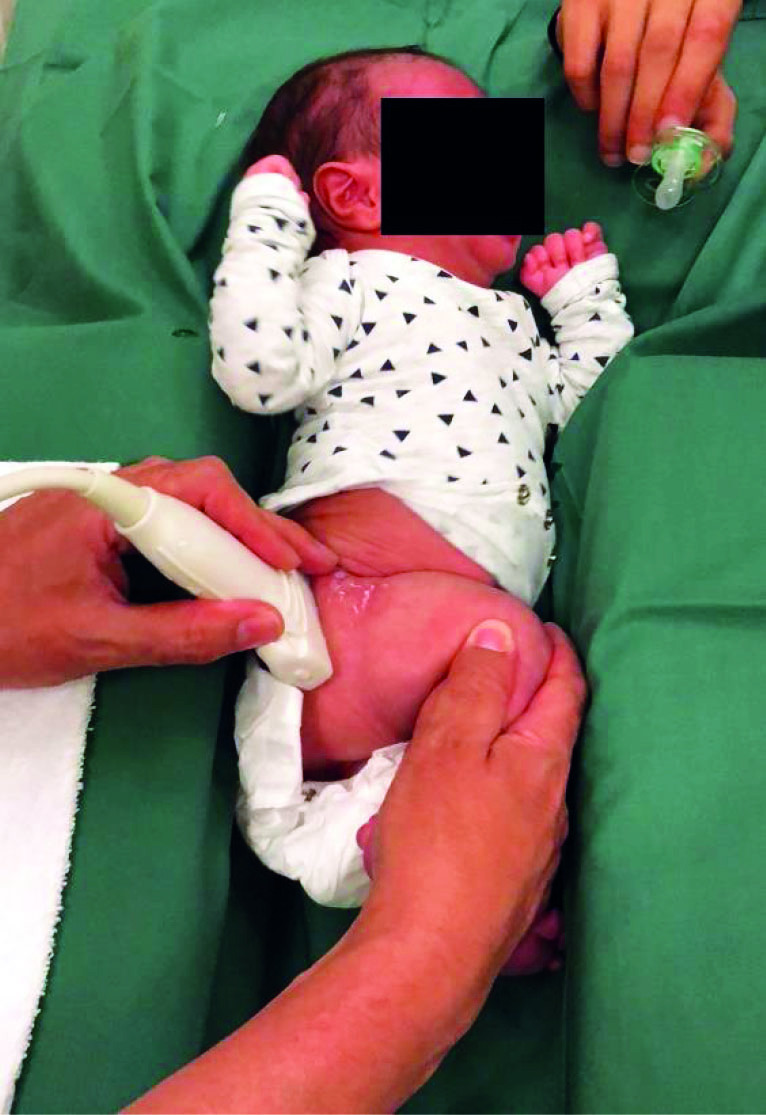
The ultrasound examination with the child in lateral position.

The ultrasound examinations were performed by experienced musculoskeletal radiologists from radiological departments in the region including our own in-house radiologists. The examination was done in the presence of the parents with a high-frequency transducer (Hitachi model: L74M 13-5; Hitachi Europe, Maidenhead, UK). According to our standard scanning protocol, a modification of the Tréguier method was used, as the child was positioned on the side as per international guidelines (AIUM 2009) (Figure 2). The PFD measurements were performed after obtaining the measurements according to the Graf method. The infant was positioned lying on its side in a specific cradle with the hip flexed in adduction, for determination of pubo-femoral distance (Tréguier et al. 2013).

The transducer was placed in a rectangular position, and thereafter slid from ventral to dorsal to visualize the bony acetabulum and rotation of the transducer to visualize the straight ilium. No inclination of the transducer is permitted as this results in bowing of the ilium and loss of hip anatomy.

**Figure 3.**
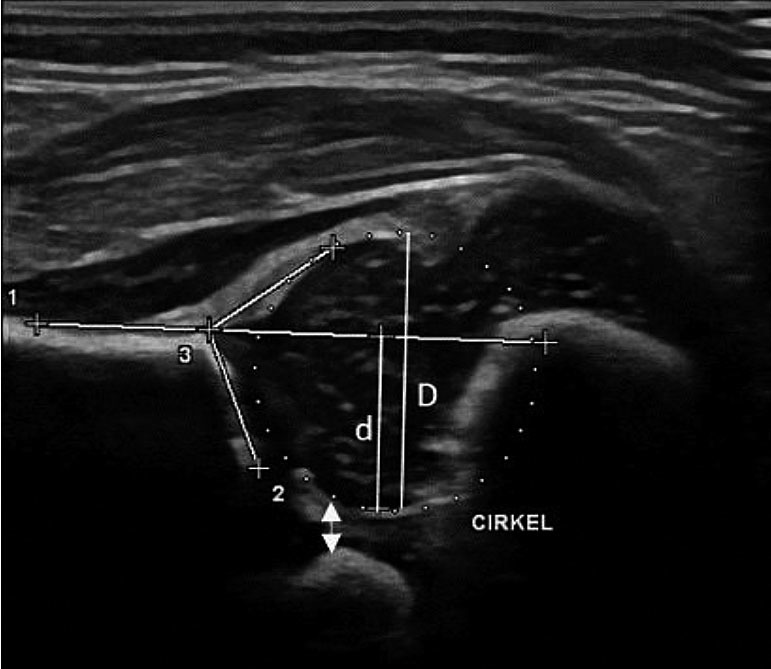
Ultrasound image of normal hip at 4 weeks. All anatomical landmarks present. The horizontal line extending from the iliac wing crosses the perpendicular femoral head diameter and defines the upper limit for d which is used to calculate BRP = d/D. The PFD is marked here by the double-headed arrow.

The quality criteria required were 2 cartilaginous landmarks consisting of the cartilaginous femoral head and the triangular hyperechoic fibrocartilaginous rim, and 3 bony landmarks consisting of the horizontal iliac wing, the bony acetabular roof at its greatest depth, and the pubic bone (Figure 3).

Hip stability was evaluated by Barlow equivalent provocation test, with the infant in the lateral position.

Alpha angles, beta angles, BRP, and PFD measurements were taken for each hip. The BRP measurement was determined by the ratio *d/D*, where *d* = the portion of the diameter of the femoral head covered by the acetabular bone, below the horizontal line extending from the iliac bone, and *D* = the (entire) femoral head diameter. PFD was measured between the medial margin of the epiphysis and the pubic bone according to the technique used by Treguier et al. (2013).

If the ultrasound detected structural abnormalities and/or hip instability was suspected, the patient would be referred to a pediatric orthopedic department for clinical testing. If DDH was confirmed, the patient would be treated with a DB brace for a minimum of 6 weeks with subsequent follow-up with repeated ultrasound examination.

### Statistics

Data were tested for normality by Shapiro–Wilk test and a paired t-test was used for comparison between groups. A p-value < 0.05 was considered statistically significant. Mean values and 95% confidence intervals (CI) are given. Receiver operating characteristic (ROC) curve analysis was done using Medcalc from MedCalc Software (https://www.medcalc.org/) according to the methodology of Delong et al. (1988) in order to evaluate specificity, sensitivity and determine an optimal cut-off point for PFD.

### Ethics, funding, and potential conflicts of interest

The Danish Data Protection Agency and the Danish Health Authority approved this single institution case-control study. The authors received no funding for this study and declare no conflicts of interest.

## Results

**Figure 4.**
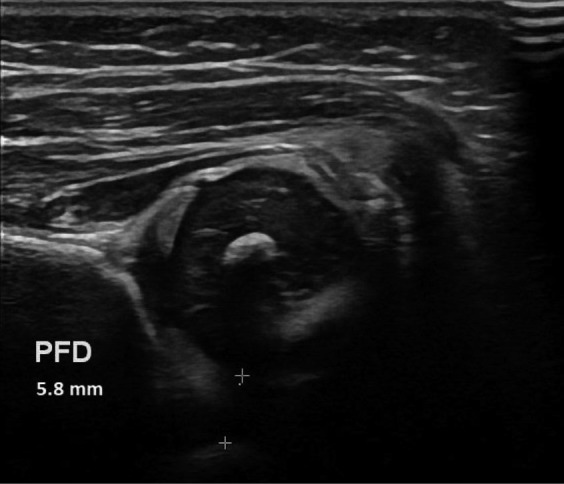
Ultrasound image of abnormal hip, girl aged 4 weeks, all anatomical landmarks present. PFD estimated at 5.8 mm as indicated by the 2 + symbols.

**Figure 5.**
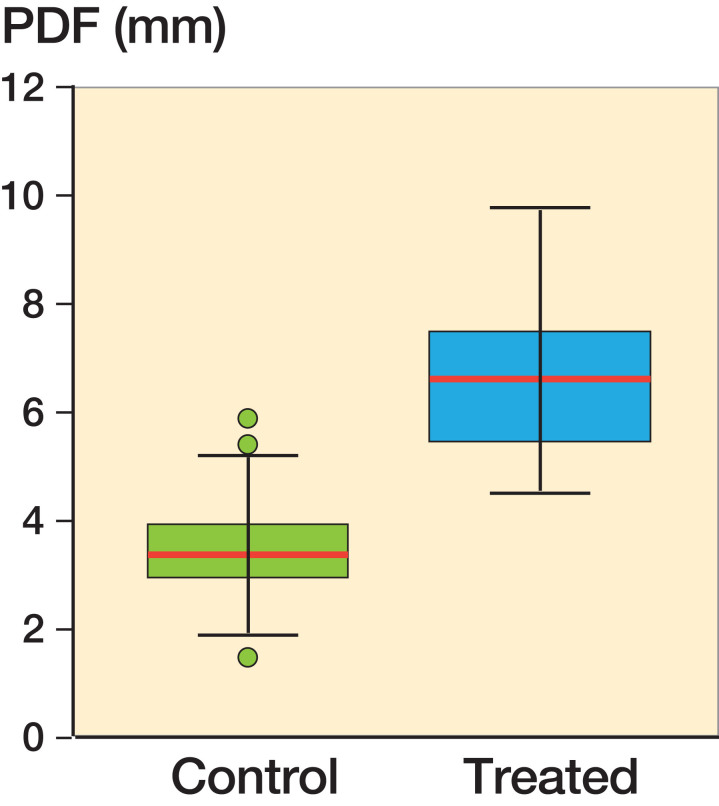
Box and whiskers plot of PFD values of controls plotted against hip with instability. The boxes represent the interquartile range. Whiskers represent the range of all values. The red line within the boxes is median value.

We found a mean PFD of 6.8 mm (CI 6.2–7.4) in the treated group compared with 3.4 mm (CI 3.3–3.6) in the controls. When compared with the control group (Table 1, Figures 4 and 5) there was a statistically significant difference for all tested parameters (alpha angle, BRP, and PFD).

**Table 1.** Alpha angles, bone rim percentage (BRP) and pubofemoral distance (PFD) of the treated and control group. Values are mean (standard deviation) [95% CI]

Group	Alpha angle (°)	p-value	BRP (%)	p-value	PFD (mm)	p-value
Controls (n = 198) Treated (n = 32)	70 (17) [68–72] 55 (5.3) [53–57]	_< 0.001_	65 (1.0) [65–66] 50 (9.7) [47–53]	_< 0.001_	3.4 (0.96) [3.3–3.6] 6.8 (1.7) [6.2–7.4]	_< 0.001_

The difference between the PFD value of the right and left hip (PFDΔ) was analyzed in both unilaterally and bilaterally affected patients (Table 2). We found a mean PFDΔ of 2.6 mm and 2.2 mm respectively. There was no statistically significant difference between PFDΔ of the unilaterally and bilaterally affected (p = 0.6), but a significant difference (p < 0.05) was found when compared with the control group.

**Table 2.** Difference in PFD between each patient’s two hips in the treated and control group. Values are mean (mm) (standard deviation) [95% CI]

	PFDΔ	p-value
Normal (n = 98)	0.47 (0.38) [0.40–0.55]	
Unilateral DDH (n = 12)	2.6 (1.3) [1.9–3.3]	< 0.001
Bilateral DDH (n = 8)	2.2 (2.3) [0.7–3.7]	< 0.001

P-value = 0.6 between unilateral DDH vs. bilateral DDH.

**Figure 6.**
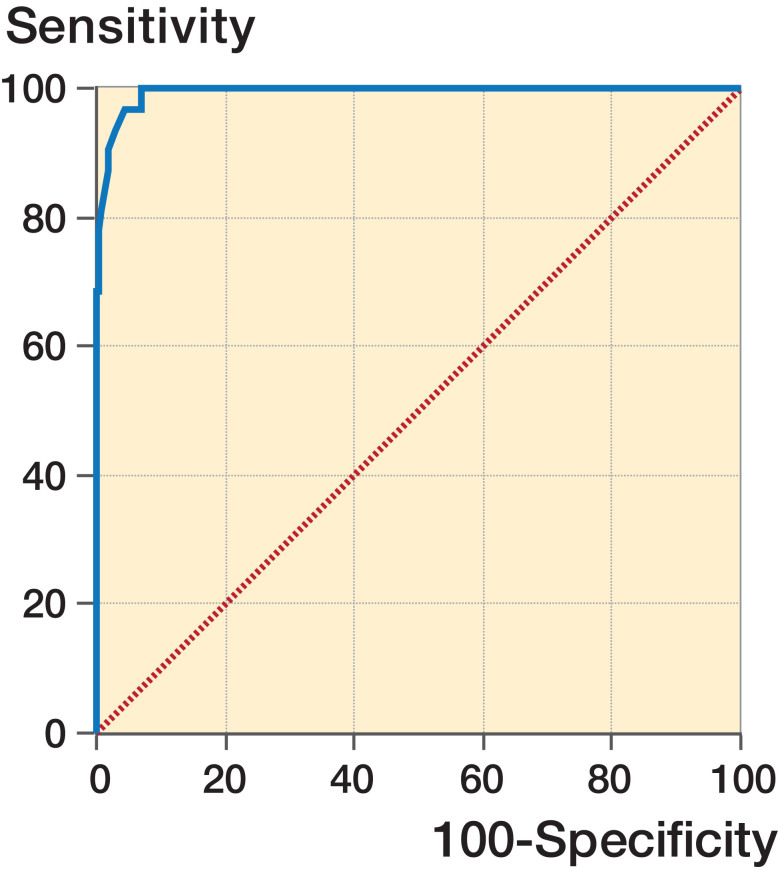
ROC graph illustrating the sensitivity and specificity for PFD in diagnosing DDH. Cut-off point was 4.4 mm. AUC = 0.99 (0.97–1.0), p < 0.001. Sensitivity was 100% and specificity was 93%.

The sensitivity was 100% and specificity 93% at a cut-off value of 4.4 mm (Figure 6).

## Discussion

The current literature on PFD measurement is sparse, even though the method has the potential to be a low-budget universal screening tool. However, the cut-off level for instability is not clear and needs to be established. Our study suggests that the cut-off value for intervention should be set lower at 4.4 mm to reach 100% sensitivity compared with 6.0 mm as previously reported. These findings may be explained by population characteristics (numbers included, hip morphology, age), different thresholds for treatment, or the fact that we used lateral position as opposed to supine as previously reported (Tréguier et al. 2013). Our findings support the findings of Teixeira et al. (2015) that a lower threshold is needed when a lateral position with adducted flexed hip is used. They found a cut-off value of 4.6 mm in their study.

We found PFD measured in lateral position to be statistically significantly increased in children with hip instability, when compared with a group of infants with normal ultrasound examinations. If diagnosis was to be made on the basis of PFD alone, all our patients treated for DDH with a DB brace would have been diagnosed and treated correctly with only 7% receiving a false-positive diagnosis.

PFD measurements have previously been shown to be very useful for assessing DDH (Couture et al. 2011, Salut et al. 2011, Tréguier et al. 2013).

Treguier et al. (2013) diagnosed DDH among 1-month old patients and found sensitivity and specificity values of 100% and 80% respectively, with a cut-off point of 6 mm in supine flexed position.

We chose to scan the infant in lateral position as all infants had Graf measurements performed as per international guidelines (AIUM 2009); this allowed both tests to be performed simultaneously and more easily.

The PFD method requires little experience by the sonographic examiner to correctly and quickly classify the patients needing intervention (Tréguier et al. 2013, Teixeira et al. 2015). The inter-observer reproducibility was tested by Treguier et al. (2013) by analyzing the level of agreement between experienced and inexperienced operators in determining the PFD and BRP classification. They found agreement in the operators’ classification in 92% of the cases for PFD and only 41% of the cases of BRP classification (Tréguier et al. 2013). The inexperienced operator used in that study was a resident radiologist in the early phase of his training, but the data may be extrapolated to other examiners such as midwives or general practitioners who have received a minimum of training in the procedure. The simplicity and the reproducibility of the method have since been confirmed and measurements have been demonstrated to be independent of hip positions (flexion or neutral) (Teixeira et al. 2015).

In contrast the Graf method can be complex and difficult to perform by inexperienced sonographers and radiologists, and requires extensive training to master (Omeroğlu et al. 2001, Hell 2008).

Rosendahl et al. (1995) compared 2 observers with 5 and 2 years of radiological experience and found a low level of agreement when evaluating each one’s image acquisition. There was a higher level of agreement when evaluating the same static images, which could indicate that the disagreement lies in the technique and obtainment of the sonographic images rather than the interpretation itself. This is supported by the distinct influence of probe positioning on the measurement results as Graf initially found himself (Graf 1983), as well as acceptable inter-observer agreement for the Graf angle measurements when performed using static images previously captured and reviewed by an experienced radiologist (Teixeira et al. 2015).

The PFD 6.0 mm threshold as diagnostic for DDH is suggested by Tréguier et al. (2015), and also a 1.5 mm difference between the 2 hips was suggested as a threshold in their paper. In our study, the differences in mean PFDΔ of affected hips when compared with the control group were found to be statistically significantly increased; however, between the hips of respectively unilaterally and bilaterally affected patients PFDΔwas found to be non-significant. This finding does not support the recommendations by Treguier et al. (2015), but this can most likely be explained by the low number of patients involved in our study and may be regarded as a type 2 error. Therefore, no firm conclusions can be made on this matter based on our study. We recommend that PFDΔ above 1.5 mm is kept as a diagnostic criterion in the lateral position until larger data-sets are available.

Our study is limited by the fact that the final impact on the rate of late diagnosis of DDH has not yet been assessed, and by its small sample population size, though we were able to demonstrate a statistically significant PFD increase in lateral position at the time of diagnosis in patients treated for DDH even with our limited number of included subjects.

In several cases, PFD measurements were not supplied in referrals from local hospitals, due to different sonographic examination standards and algorithms, and in some cases this was not performed by in-house radiologists due to dislocation of the examined hip, thus reducing the amount of data available for analysis.

Dislocation of the hip makes the PFD immeasurably high and it would therefore exceed our proposed cut-off point, as well as producing a positive clinical examination, and the patient would therefore be referred for specialized ultrasound on both grounds.

Since this method was introduced to the screening program of 2 million people in the French population by Tréguier et al. (2013), they have reported a 75% reduction in the rate of late diagnosis. In order to completely eradicate late DDH diagnoses, they propose a universal screening program for girls, as they have found that 70% of infants put into traction for DDH are girls, and this itself is a risk factor for DDH. This finding is supported by data from other groups (Marks et al. 1994, Paton et al. 2005, Vane et al. 2005) and our study where 23/25 of treated patients were girls.

Clinical examination alone is insufficient in diagnosing DDH in infants as mild acetabular dysplasia and instability are common, and even selective ultrasound screening has been found to be inadequate when using current screening criteria (Sink et al. 2014). This prompts the need for a more generalized screening program for DDH, which would require a sensitive, easily learned and low-cost method of examination. We believe the PFD measurement fulfills these criteria for the reasons listed above and that it could become a future referral criterion for referral for the specialized Graf US examination.

A primary concern of the application of universal screening of female infants is the risk of generating a high number of false-positives. This, combined with the fact that the PFD measurement in some cases is seen to be increased above the diagnostic threshold due to thickening of the pubic bone cartilage (Tréguier et al. 2013) in otherwise clinically normal and stable hips in healthy infants, is a concern. However, since the implementation of the screening program in their female population, Treguier et al. has not seen a significant increase in consultation or splintage rates.

In order to further evaluate PFD measurements as a referral criterion in the screening algorithm for DDH in Denmark in the future, a study with a prospective design, and consistent use of the measurements in all referred patients—both primary referrals and secondary referrals from other hospitals—is necessary.

In summary, PFD in lateral position was shown to be significantly increased in hips of children treated for DDH with DB brace compared with healthy children with unaffected stable hips. A cut-off value of 6.0 mm has previously been reported as optimal in supine position. We suggest that 4.4 mm is used in lateral position. PFD may be a feasible low-cost tool for universal screening of infants and could be a supplement to the current highly specialized examination of risk groups in Denmark.

HH, OR, and MH planned the paper. HH drafted the manuscript and performed statistical analyses. All authors revised and approved the final manuscript

*Acta* thanks Deborah M Eastwood and Ola Wiig for help with peer review of this study.

AIUM. Practice guideline for the performance of an ultrasound examination for detection and assessment of developmental dysplasia of the hip. J Ultrasound Med 2009; 28: 114-19.
